# Beyond the Phenothiazine Core: Mechanistic Insights into the Three-Electron Oxidation of Chlorpromazine

**DOI:** 10.3390/molecules30051050

**Published:** 2025-02-25

**Authors:** Kiara T. Miller, Ashwin K. V. Mruthunjaya, Angel A. J. Torriero

**Affiliations:** School of Life and Environmental Sciences, Faculty of Science Engineering & Built Environment, Deakin University, Burwood, VIC 3125, Australia

**Keywords:** electrochemical oxidation, chlorpromazine, phenothiazine, spectroelectrochemistry, CPZ sulfoxide, nor-CPZ sulfoxide

## Abstract

This study investigates the electrochemical oxidation mechanisms of chlorpromazine (CPZ), revealing a novel three-electron oxidation pathway that challenges the traditionally accepted two-electron paradigm, offering new insights into CPZ oxidation pathways. Using an integrated approach combining cyclic voltammetry, bulk electrolysis, UV-Vis, FT-IR, ^1^H-NMR spectroscopy, and LC-MS/MS analysis, we demonstrate that CPZ undergoes sequential oxidation processes involving both the phenothiazine core and the tertiary amine-containing side chain. Our results highlight the critical role of side-chain oxidation in forming nor-CPZ sulfoxide, an often-overlooked metabolite, which may influence CPZ’s metabolic and pharmacological behaviour. Spectroelectrochemical data reveal stable intermediate species, providing insight into the structural rearrangements accompanying oxidation. This work offers a detailed mechanistic understanding of CPZ redox behaviour, contributing to improved interpretations of its pharmacological and metabolic properties.

## 1. Introduction

Chlorpromazine (CPZ), a phenothiazine (PTZ) derivative, was introduced in the 1950s and remains a cornerstone in the treatment of psychotic disorders such as schizophrenia [1,2]. As one of the first antipsychotic drugs, CPZ revolutionised psychiatric care by targeting dopamine D2 receptors while also interacting with serotonin, histamine, and adrenergic receptors, contributing to its broad pharmacological effects [3,4,5]. Beyond its psychiatric applications, CPZ has been explored for antimicrobial and oncological uses, where oxidative mechanisms may influence its activity and metabolism [6,7]. Despite its broad therapeutic role, CPZ is associated with significant side effects, including movement disorders and hepatic toxicity, which are attributed to its metabolic and oxidative pathways [8,9]. Understanding the electrochemical oxidation of CPZ is thus essential for elucidating its pharmacological mechanisms and developing strategies to optimise therapeutic outcomes while minimising adverse effects.

The oxidative mechanisms of CPZ, as reported in the literature over the last 60 years, have largely mirrored those of the parent PTZ structure [10,11,12,13,14]. Most studies have proposed a two-electron mechanism involving the sequential oxidation of the nitrogen and sulphur atoms in the tricyclic PTZ core [15,16,17]. These assumptions have been primarily based on electrochemical studies using cyclic voltammetry in aqueous systems, where oxidation peaks were attributed to processes occurring solely within the core structure [15,16,17,18,19,20,21,22,23,24]. However, this approach fails to account for the unique structural features of CPZ, particularly its tertiary amine-containing side chain—an aspect often overlooked in prior studies, likely due to the predominant focus on the PTZ core and the limited use of techniques capable of detecting side-chain oxidation. This oversight has led to an incomplete understanding of the CPZ oxidation mechanism, restricting the correlation between electrochemical findings and its biological metabolism. Additionally, the behaviour of CPZ in organic solvents remains underexplored, presenting an opportunity to expand our understanding of its redox properties.

A critical examination of past studies reveals further limitations in the mechanistic interpretations of CPZ oxidation. For instance, many studies have reported the immediate formation of CPZ sulfoxide as a direct product of sulphur oxidation without considering the kinetics of its formation or the role of intermediate species [19,25,26]. Liquid chromatography–mass spectrometry (LC-MS) studies have been employed to identify sulfoxide and other metabolites; however, they often rely heavily on mechanisms proposed for PTZ oxidation, neglecting the specific structural features of CPZ [19,25]. For example, while such studies confirm the formation of CPZ sulfoxide, they fail to account for the delayed appearance of this metabolite under certain conditions or the role of side-chain oxidation. Additionally, mechanistic interpretations often lack spectroelectrochemical insights to track transient intermediates, which are critical for understanding the progression of oxidation. This gap has led to incomplete or misleading conclusions about CPZ’s redox pathways. Moreover, the assumption that CPZ oxidation mirrors that of PTZ disregards the potential for side-chain oxidation, which may contribute to the formation of metabolites such as nor-CPZ sulfoxide—an oxidation metabolite commonly detected in human urine samples and associated with the drug’s metabolic pathway [27,28]. These gaps highlight the need for a more nuanced understanding of CPZ’s redox pathways, integrating core and side-chain contributions.

Another notable gap in the literature is the need for comprehensive spectroscopic validation of the proposed mechanisms. While cyclic voltammetry provides valuable insights into redox processes, it alone cannot provide structural information. It only indicates the presence and lifetime of intermediates when they are electroactive and within the redox potential window studied. Ultraviolet-visible (UV-Vis) and Fourier-transform infrared (FT-IR) spectroscopies have been used sporadically to complement electrochemical data. Yet, their application on PTZ and derivatives has often been restricted to confirming end products rather than probing intermediate species [29,30]. These methodological limitations have perpetuated a narrow focus on the phenothiazine core, neglecting the complexity introduced by the side chain and other functional groups.

This paper addresses these critical gaps by presenting a comprehensive study of CPZ oxidation using advanced electrochemical and spectroscopic techniques. Although the electrochemical oxidation of CPZ in acetonitrile exhibits multiple peaks [25], this study focuses on the first two oxidation processes, as they are critical for understanding the initial mechanistic pathways and redox behaviour of the molecule. By limiting the scope to these initial processes, we aim to provide a clear and detailed characterisation of CPZ’s redox properties without being confounded by the complexity of subsequent oxidation events. For example, this work establishes a three-electron oxidation mechanism for CPZ for the first time, challenging the long-standing two-electron paradigm. By integrating controlled-potential bulk electrolysis (BE) with UV-Vis, FT-IR, proton-nuclear magnetic resonance (^1^H-NMR), and LC-MS/MS, this study provides a qualitative, detailed temporal, and structural analysis of the intermediates and products formed during CPZ oxidation. The findings reveal that CPZ sulfoxide does not form immediately upon oxidation in acetonitrile but instead emerges over time, underscoring the importance of in situ studies. Additionally, the detection of nor-CPZ sulfoxide highlights the role of side-chain oxidation, a process often overlooked in earlier research. This work emphasises the qualitative nature of the results, focusing on identifying and characterising intermediates and reaction pathways, thereby setting a new standard for investigating the oxidative mechanisms of phenothiazine derivatives. While these findings may inform future studies on therapeutic optimisation and drug design, this study does not directly address biological or pharmacological responses.

## 2. Results and Discussion

The electrochemical oxidation of PTZ has been extensively studied and is often used as a reference system to understand the redox properties of its derivatives. Historically, PTZ oxidation has been experimentally described as two sequential one-electron transfer steps [10,12], classified as an EE mechanism, where “E” refers to an electrochemical electron transfer. This mechanism was validated through electrochemical studies that identified radical cation (PTZ^•+^) and dication (PTZ^2+^, Equation (1), were R=H for PTZ or an alkyl group for PTZ derivatives) intermediates under specific conditions [10,12]. However, some studies suggest that this mechanism is often oversimplified and does not fully account for follow-up chemical reactions (C) that can occur after the second electron transfer [14,15].(1)
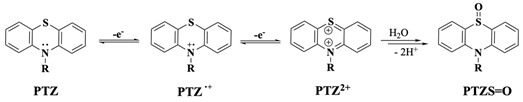


**Figure 1:**
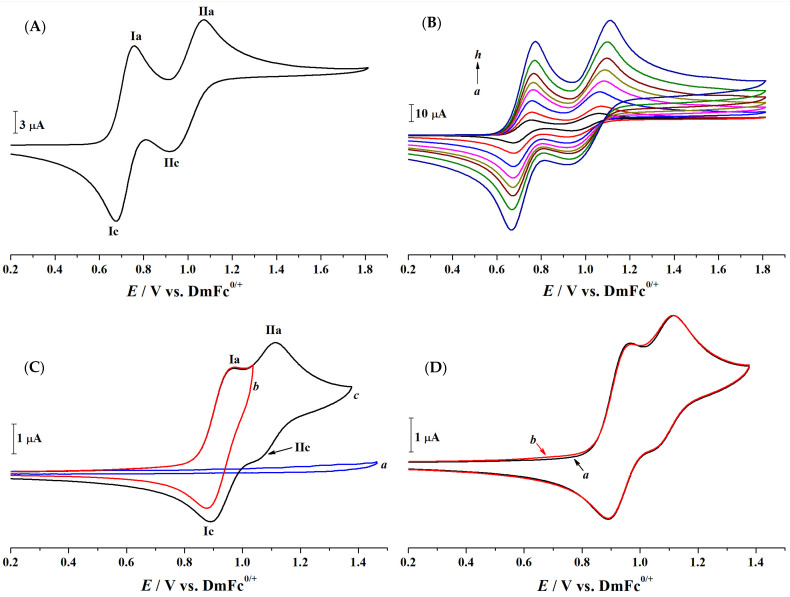
Cyclic voltammograms recorded at a 1.0 mm diameter GC electrode in CH_3_CN containing 0.1 M [Bu_4_N][PF_6_] as the supporting electrolyte: (**A**) at a scan rate of 0.1 Vs^−1^ in the presence of 2.61 mM PTZ; (**B**) in the presence of 2.61 mM PTZ at scan rates of (*a*) 0.05, (*b*) 0.10, (*c*) 0.20, (*d*) 0.30, (*e*) 0.40, (*f*) 0.50, (*g*) 0.70, and (*h*) 1.00 Vs^−1^; (**C**) at a scan rate of 0.1 Vs^−1^ in the absence (*a*) and presence (*b*, *c*) of 1.27 mM CPZ, with switching potentials of (*a*) 1.46, (*b*) 1.04, and (*c*) 1.38 V vs. DmFc^0/+^; and (**D**) at a scan rate of 0.1 Vs^−1^ in the presence of 1.27 mM CPZ under air-saturated conditions (*a*) and nitrogen (*b*) atmosphere. T = 21 ± 1 °C.

The cyclic voltammograms of PTZ, presented in Figure 1A,B, provide insights into its oxidation mechanism under our experimental conditions. The first oxidation process (Figure 1A, peak I) appears at a mid-point potential (*E*_m_, calculated from the average of the oxidation ($E_{p_{Ia}}$) and reduction ($E_{p_{Ic}}$) peak potentials, $E_{m(I)}=(E_{p_{Ia}}+E_{p_{Ic}})/2)$ of 0.716 V versus the DmFc^0/+^ IRS, with a peak-to-peak separation of 78 mV, consistent with a reversible one-electron process. This behaviour aligns with previous findings, where PTZ^•+^ was identified as the primary oxidation product [12,31]. The second process (Figure 1A, peak II), occurring at *E*_m(II)_ of 0.996 V, exhibits a peak-to-peak separation of 150 mV, characteristic of a quasi-reversible electron transfer. Furthermore, the broader and rounded shape of process IIc, which is easily observed at fast scan rates (Figure 1B), suggests that follow-up reactions complicate the interpretation of this process.

Previous studies propose two main types of follow-up reactions after the formation of PTZ^2+^. The first suggests interaction with water molecules, forming a sulfoxide product (PTZS=O), as depicted in Equation (1) [11,14]. Others have reported that dimerisation reactions can occur under certain experimental conditions [10,11,15]. These follow-up chemical transformations shift the mechanism from a simple EE process to an EEC or even an EECC pathway, depending on the experimental environment and the species involved. Such observations underscore the complexity of PTZ oxidation mechanisms, which rely heavily on the experimental conditions. For example, when PTZ is oxidised in non-aqueous environments like sulphur dioxide, the PTZ oxidation was described as purely an EE mechanism with no chemical follow-up reactions detected under their specific conditions [12]. However, follow-up reactions like sulfoxide formation or dimerisation become evident when aqueous or mixed solvents are used [10,11,14,15].

Figure 1C,D show the cyclic voltammograms of 1.27 mM CPZ in CH_3_CN containing 0.1 M [Bu_4_N][PF_6_] as the supporting electrolyte. Process I, corresponding to the first electron transfer, occurs at an *E*_m(I)_ of 0.931 V versus the DmFc^0/+^ IRS. This value is positively shifted compared to the *E*_m(I)_ of 0.716 V observed for PTZ (Figure 1A). This shift results from the combined effects of the substituents on the CPZ structure. The chlorine atom, being electron-withdrawing, stabilises the molecular orbitals of the starting material, leading to an increase in the oxidation potential of CPZ compared to PTZ. While alkyl groups in the tertiary amine exhibit an electron-donating inductive effect, their contribution is less dominant. Such substituent effects have been consistently observed in phenothiazine derivatives [13,32]. Process II, associated with the second CPZ oxidation step, exhibits a positive potential shift compared to PTZ. Although the exact *E*_m_ position of process II is challenging to determine with precision, it is about 0.150 V more positive than process I. The shift in both oxidation processes reflects the combined effects of substituent-induced electronic modifications to the PTZ core.

Figure 1D illustrates the cyclic voltammograms of 1.27 mM CPZ in CH_3_CN containing 0.1 M [Bu_4_N][PF_6_] as the supporting electrolyte in the presence and absence of oxygen. The voltammograms are identical under both conditions, indicating that the redox processes of CPZ are unaffected by the presence of oxygen. This result demonstrates that oxygen does not participate in or interfere with the electrochemical oxidation of CPZ under the experimental conditions employed. Consequently, all subsequent experiments were conducted under air-saturated conditions, simplifying the experimental setup without compromising the accuracy or validity of the results.

**Figure 2:**
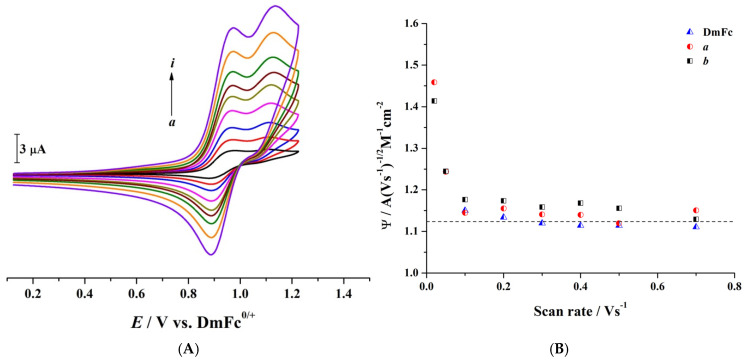
(**A**) Cyclic voltammograms of 1.65 mM CPZ in CH_3_CN containing 0.1 M [Bu_4_N][PF_6_] as the supporting electrolyte, recorded at a 1.0 mm diameter GC electrode and scan rates of (*a*) 0.02, (*b*) 0.05, (*c)* 0.10, (*d*) 0.20, (*e*) 0.30, (*f)* 0.40, (*g*) 0.50, (*h*) 0.70, and (*i*) 1.00 Vs^−1^. (**B**) Dependence of current function (Ψ) on scan rate for CPZ under nitrogen (*a*) and air (*b*) atmosphere. The DmFc^0/+^ process is used as the reversible one-electron transfer reference compound. T = 21 ± 1 °C.

Figure 2A presents the cyclic voltammograms of 1.65 mM CPZ at varying scan rates. From these voltammograms, the experimental current function (Ψ) was determined and plotted against the potential scan rate (Figure 2B) to provide insights into the electrochemical oxidation mechanism of CPZ. The Ψ, defined as Ψ = *I*_p(Ia)_/(A × *c* × *v*^1/2^) (where *I*_p(Ia)_ is the CPZ peak current of process Ia, A is the working electrode area, *c* is the bulk concentration of CPZ, and *v* is the scan rate) was used to determine the “apparent” number of electrons exchanged (n_app_) associated with the oxidation process I by comparing its value with that of DmFc, which was employed as a one-electron reference system. This estimation assumes that CPZ and DmFc have similar or comparable diffusion coefficients, and the data are obtained under identical experimental conditions [33].

At higher scan rates (≥0.1 Vs^−1^), Ψ of CPZ has a value comparable to that of DmFc (1.11 A (V s^−1^)^−1/2^ M^−1^ cm^−2^), indicating that the initial oxidation of CPZ to its radical cation (CPZ^•+^) is a simple, reversible one-electron transfer. However, as the scan rate decreases below 0.1 Vs^−1^, an increase in Ψ is observed, implying the involvement of additional electron transfer steps. This scan rate dependence indicates that while the initial electron transfer is rapid and reversible, preceding or subsequent chemical reactions or structural reorganisations may become significant at lower scan rates, leading to an overall increase in the number of electrons transferred.

**Figure 3:**
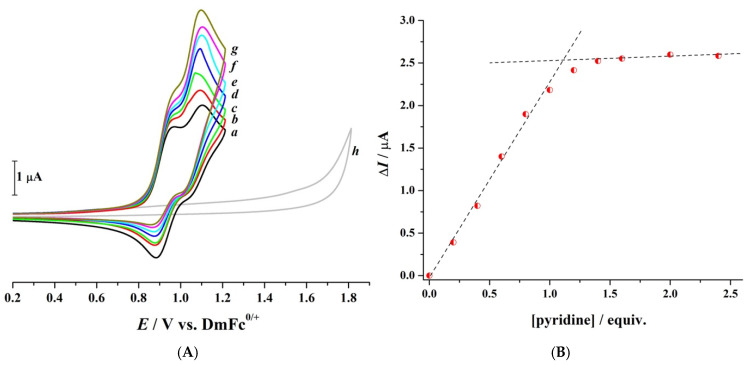
Electrochemical data obtained for oxidation of 1.0 mM CPZ in CH_3_CN containing 0.1 M [Bu_4_N][PF_6_] as the supporting electrolyte in the absence and presence of pyridine: (**A**) cyclic voltammograms recorded at 1.0 mm diameter GC electrode at a scan rate of 0.10 Vs^−1^ in the absence (*a*) and presence of (*b)* 0.2, (*c*) 0.4, (*d*) 0.6, (*e*) 0.8, (*f*) 1.0, (*g*) 1.6 equivalents of pyridine, and (*h*) 0.2 mM pyridine in the absence of CPZ; and (**B**) dependence of current change (∆*I* = peak current of process II obtained from cyclic voltammetry) for CPZ upon addition of designated concentrations of pyridine. T = 21 ± 1 °C.

To further investigate the electrochemical behaviour of CPZ and elucidate the role of its protonated tertiary amine group in the increase in Ψ observed at low scan rates, a titration experiment was conducted by incrementally adding pyridine to a 1.65 mM CPZ solution (Figure 3). Pyridine, a known base, deprotonates the protonated tertiary amine of CPZ.HCl (Equation (2)), allowing for the assessment of its influence on the electrochemical responses.(2)
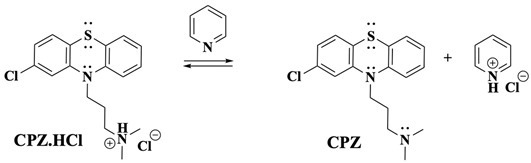


As pyridine was added, a notable increase in the current associated with process II was observed (Figure 3), reaching a maximum upon adding one equivalent of pyridine. This equivalence point is expected for a single deprotonation step in an acid-base titration. Minor deviations (e.g., ~1.15 equivalents) may arise due to minor experimental variations, but they do not impact the mechanistic interpretation. This saturation behaviour suggests that the observed electrochemical changes are linked to a distinct molecular transition occurring at this stoichiometric ratio. Two possible scenarios may explain this current behaviour upon reaching the equivalence point: (*i*) an enhancement of the existing process II due to the deprotonation of the tertiary amine, facilitating electron transfer, or (*ii*) the emergence of a new process associated with the direct oxidation of the tertiary amine, which overlaps with process II. Tertiary amines are well-documented to oxidise within this potential range, as supported by the literature on the oxidation of aliphatic and aromatic amines, where the amine’s lone pair of electrons and structural features significantly influence their oxidation potential [34]. Thus, besides the PTZ core of CPZ, the side-chain tertiary amine is likely contributing to the observed electrochemical behaviour. Control experiments were conducted to ensure that the changes observed were specific to interactions between CPZ.HCl and pyridine. Cyclic voltammogram “*h*” in Figure 3A demonstrates that pyridine is electrochemically inactive within the studied potential window, confirming that the observed changes are due to CPZ and not intrinsic pyridine activity.

To further elucidate the redox mechanisms associated with CPZ processes I and II, controlled-potential bulk electrolysis (BE) experiments were conducted in CH_3_CN (0.1 M [Bu_4_N][PF_6_]) solutions. The first BE was performed at an applied potential (*E*_app_) of 0.950 V vs. DmFc^0/+^, a potential just below the peak maximum for process I, thereby ensuring selective oxidation of process I due to its proximity to process II. Coulometric analysis revealed an apparent number of electrons transferred (n_app_) of 2.0 ± 0.2 electrons per molecule of CPZ, suggesting a two-electron process. A second BE experiment, conducted at *E*_app_ = 1.250 V vs. DmFc^0/+^, a potential beyond process II, yielded a total n_app_ of 3.0 ± 0.2 electrons per molecule of CPZ. This value confirms the sequential nature of the redox processes, with process II involving an additional one-electron transfer following the initial two-electron process observed in process I.

Analogous BE experiments were conducted on PTZ, the parent structure of CPZ, to validate these findings. The first BE experiment was performed at *E*_app_ = 0.758 V vs. DmFc^0/+^, corresponding to the oxidation peak potential of process I for PTZ. The coulometric analysis for this step revealed an n_app_ = 1.0 ± 0.1 electrons per PTZ molecule, consistent with previous literature [10,11,14]. A subsequent BE experiment at *E*_app_ = 1.200 V vs. DmFc^0/+^, corresponding to process II, indicated n_app_ = 2.0 ± 0.1 electrons per molecule, further corroborating earlier reports [10,11,14].

**Figure 4:**
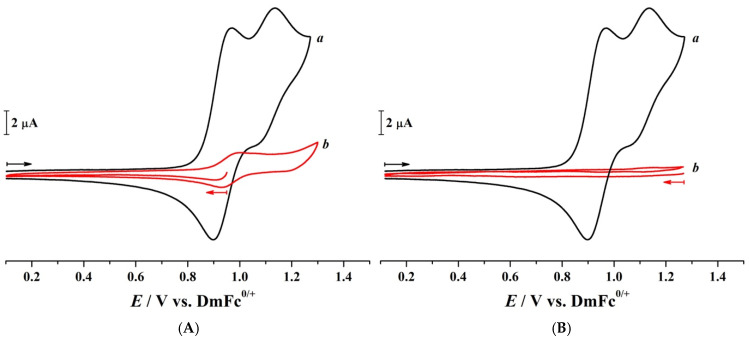
Cyclic voltammograms of 4.74 mM CPZ in CH_3_CN containing 0.1 M [Bu_4_N][PF_6_] as the supporting electrolyte obtained before (*a*) and after (*b*) exhaustive controlled-potential bulk electrolysis at (**A**) *E*_app_ = 0.950 V vs. DmFc^0/+^ and (**B**) *E*_app_ = 1.250 V vs. DmFc^0/+^. The arrows indicate the potential scan direction. Scan rate = 0.10 Vs^−1^ T = 21 ± 1 °C.

To verify reproducibility and gain deeper insights into the underlying redox events, the CPZ bulk electrolysis experiments were repeated, and CVs were recorded before and after each BE at selected potentials (Figure 4). After each BE was completed, the CV measurement commenced at the *E*_app_. It was subsequently scanned in the negative potential direction, allowing for the detection of any oxidised species remaining in the solution. By comparing the outcomes of BE at *E*_app_ = 0.950 and 1.250 V vs. DmFc^0/+^, distinct electrochemical profiles emerged. Following BE at 0.950 V, a small yet reversible intermediate species with an *E*_m_ of 0.967 V vs. DmFc^0/+^ was detected, indicating the formation of an electroactive intermediate. Conversely, after BE at 1.250 V, no residual electroactive signals remained, confirming the exhaustive oxidation of CPZ and its intermediates. Interestingly, the n_app_ = 2.0 observed for process I of CPZ aligns with the elevated Ψ detected at slower scan rates (Figure 2B), indicating that additional kinetically hindered steps, which may involve a deprotonation event before electron transfer, could be contributing to the overall redox process.

**Figure 5:**
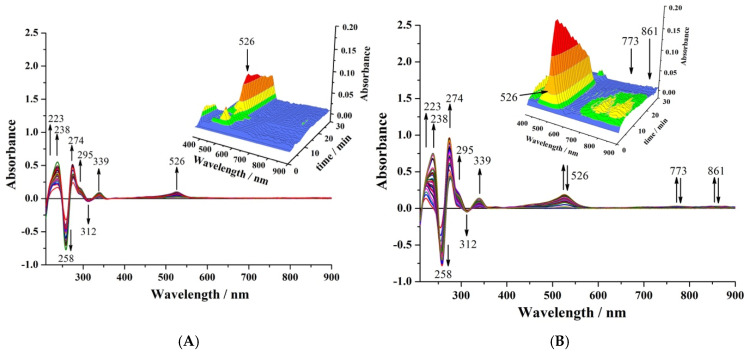
UV-Vis spectroelectrochemical experiment for oxidation of 0.39 mM CPZ in CH_3_CN containing 0.1 M [Bu_4_N][PF_6_] as the supporting electrolyte: scans recorded every 1 min during electrolysis at (**A**) *E*_app_ = 0.950 V vs. DmFc^0/+^ and (**B**) *E*_app_ = 1.250 V vs. DmFc^0/+^. T = 21 ± 1 °C. Inset: 3D plot of data in the 350–900 nm range that enhances the bands at 526, 773, and 861 nm.

Figure 5 illustrates the UV-Vis spectroelectrochemical analysis of CPZ oxidation in CH_3_CN, showcasing the dynamic changes in electronic transitions during the stepwise oxidation process using 0.1 M [Bu_4_N][PF_6_] as the supporting electrolyte. The subtractive method was employed to collect spectral data, a less commonly used but highly effective approach for identifying changes in electronic transitions during electrochemical processes. In this method, spectra recorded during the oxidation process are subtracted from the spectrum of the initial state, allowing the visualisation of both positive and negative peaks. Negative peaks correspond to the disappearance of electronic transitions associated with the starting material (CPZ), while positive peaks signify the appearance of new transitions related to oxidised species.

The subtractive spectra revealed two prominent negative peaks at 258 and 312 nm, corresponding to the electronic transitions of the starting CPZ molecule. The peak at 258 nm is assigned to a π-π* transition within the conjugated aromatic system of the PTZ nucleus, a feature characteristic of highly conjugated aromatic systems [35]. The 312 nm peak is attributed to an n-π* transition involving the nonbonding electrons of the nitrogen atom in the thiazine ring, consistent with transitions observed in PTZ derivatives [36]. These transitions disappear upon oxidation as the electron density on the conjugated system and heteroatoms decreases, altering the energy levels of the molecular orbitals and quenching these absorptions.

Figure 5A corresponds to the oxidation of CPZ at 0.950 V vs. DmFc^0/+^. This reaction is marked by the appearance of several positive peaks at 223, 238, 274, 295, 339, 413, 440, 467, and 526 nm, reflecting new electronic transitions associated with CPZ^•+^. The peaks at 223 and 238 nm are attributed to π-π* transitions in the aromatic phenothiazine system, slightly shifted due to the altered electronic structure of the radical cation. The peaks at 274 nm and 295 nm are similarly assigned to modified π-π* transitions within the phenothiazine ring [35].

The peak at 339 nm represents either a charge-transfer (CT) transition or a π-π* excitation involving weak delocalisation of the unpaired electron across the aromatic rings. In the visible region, the peaks at 413 nm, 440 nm, and 467 nm are characteristic of CT transitions within the radical cation, where the unpaired electron interacts weakly with the PTZ’s conjugated system. Finally, the distinct peak at 526 nm is identified as a low-energy electronic transition unique to the radical cation, reflecting the weak delocalisation of the unpaired electron. As observed in Figure 5A, this signal persists throughout the UV-Vis spectroelectrochemical experiment, indicating the stability of the intermediate species generating this peak. This observation aligns with the findings from Figure 4A, where a stable electroactive intermediate is detected at the end of the BE, further supporting the persistence and stability of this intermediate. However, as shown in Figure 5B, the 526 nm signal disappears when an *E*_app_ of 1.250 V vs. DmFc^0/+^ is used during the UV-Vis spectroelectrochemical experiment. This electronic transition fades entirely after 20 min, suggesting that the intermediate is fully consumed upon further oxidation, forming the final oxidised species. Based on Figure 5A, the radical cation is stable and persists throughout the bulk electrolysis experiment for process I, ruling out the possibility of follow-up reactions contributing to its disappearance. This assignment aligns well with previous findings, where a similar transition for phenothiazine radical cations was observed, emphasising the contribution of the sulphur lone pairs to the singly occupied molecular orbital (SOMO) orbital in stabilising the radical species [35].

Figure 5B shows the UV-Vis spectroelectrochemical analysis of CPZ when an *E*_app_ more positive than that for process II is used. In addition to retaining all the positive peaks observed in process I, two new peaks emerge at 773 nm and 861 nm, which are characteristic of low-energy electronic transitions. These transitions are attributed to extensive charge delocalisation over the planarised cationic structure, involving the sulphur and nitrogen atoms as well as the aromatic π-system. The planarisation of CPZ^2+^, driven by the increased electrostatic repulsion between the two positive charges, enhances conjugation and allows sulphur’s lone pairs to participate more heavily in charge stabilisation and electronic transitions.

The peaks at 773 nm and 861 nm, observed during process II, reflect long-wavelength CT transitions resulting from charge redistribution across a fully delocalised cationic system. This behaviour aligns with EPR studies of phenothiazine derivatives, suggesting a bent geometry for CPZ and its radical cation forms, which transition to a planar structure when CPZ^2+^ is formed [36,37]. These conformational changes, supported by detailed EPR studies [37], enhance delocalisation and stabilise electronic reorganisation, leading to the emergence of these near-infrared, NIR, peaks. However, as shown in Figure 5B, these peaks disappear after 20 min of BE, indicating the consumption of CPZ^2+^, potentially through slow follow-up reactions.

The role of sulphur in stabilising CPZ^•+^ and CPZ^2+^ is particularly interesting. While older EPR studies emphasised nitrogen as the primary site of unpaired electron density in radical cations and partially distributed across the aromatic ring [38,39], a recent study highlighted the significant contribution of sulphur to the SOMO orbital in PTZ systems [35]. The observed UV-Vis transitions, particularly the 526 nm peak in CPZ^•+^ and the NIR peaks in CPZ^2+^, strongly support Noda’s findings. Despite being partially electron-deficient in the oxidised states, these spectral features suggest that sulphur facilitates charge delocalisation and enables low-energy transitions through its interaction with the aromatic π-system.

**Figure 6:**
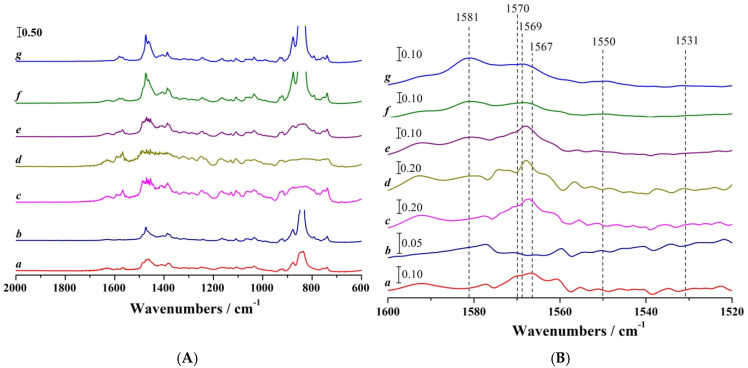
FT-IR spectroelectrochemical monitoring of the oxidation of 60 mM CPZ in CH_3_CN containing 0.1 M [Bu_4_N][PF_6_] as the supporting electrolyte. Samples of BE solution were drop-cast on KBr disks. (**A**) Full FT-IR spectra recorded before and during BE at *E*_app_ = 1.250 V vs. DmFc^0/+^, T = 21 ± 1 °C: (*a*) spectra of CPZ mixed with [Bu_4_N][PF_6_] and (*b*) [Bu_4_N][PF_6_] recorded before BE, (*c*–*f*) correspond to spectra recorded after 1, 3, 5, and 10 min of BE, respectively, and (*g*) represents the spectrum at the end of the BE. (**B**) Expanded view of the 1600–1520 cm^−1^ region from the spectra shown in (A).

To further investigate the structural transformations proposed from the UV-Vis spectroelectrochemistry study, where the formation of C=N and C=S bonds was suggested as key contributors to the observed electronic transitions and planarisation of the oxidised structure, FT-IR spectroelectrochemical analysis was performed. Figure 6 presents the FT-IR spectra of CPZ recorded before and during BE, capturing the vibrational changes associated with the oxidation process. The spectral evolution highlights distinct features in the 1600–1520 cm^−1^ region (Figure 6B), which provide evidence for specific bond formations and structural rearrangements. The FT-IR spectra of CPZ before BE starts show two prominent signals at 1570 and 1567 cm^−1^, which gradually disappear as the BE progresses. These peaks are associated with aromatic C=C stretching vibrations within the phenothiazine ring system [40]. Their disappearance indicates structural and electronic reorganisation of the aromatic system upon oxidation. Concurrently, new signals emerge in the spectrum, with key vibrational bands observed at 1581, 1569, 1550, and 1531 cm^−1^. The peak at 1581 cm^−1^ corresponds to C=C stretching vibrations within the oxidised aromatic system, reflecting increased conjugation and structural rigidity due to oxidation [40]. The appearance of the 1569 cm^−1^ band, following the disappearance of the 1570 and 1567 cm^−1^ signals, suggests an aromatic C=C stretching vibration in the oxidised phenothiazine ring, stabilised by resonance and structural rearrangement [41]. Meanwhile, the signal at 1550 cm^−1^ is attributed to C=N stretching vibrations, where the nitrogen carries a net positive charge as part of a quaternary-like structure. This positive charge significantly withdraws electron density from the double bond, stiffening the bond and causing a shift to a higher frequency compared to neutral imine groups. The 1531 cm^−1^ signal corresponds to C-N stretching vibrations, with potential contributions from C=S stretching, suggesting the presence of thiocarbonyl-like groups in the oxidation product.

Importantly, no signals were detected in the 1030–1070 cm^−1^ range, where S=O stretching vibrations are typically observed [42]. This absence indicates that sulphur does not predominantly form sulfoxide bonds (S=O) during oxidation. Furthermore, the lack of signals in the 1080–1090 cm^−1^ range suggests that no donative S–O bonds are formed. Instead, the C=S bond, coupled with the observed C=N functionalities, aligns with a planar structure post-oxidation, supporting the conclusions from UV-Vis spectroscopy.

**Figure 7:**
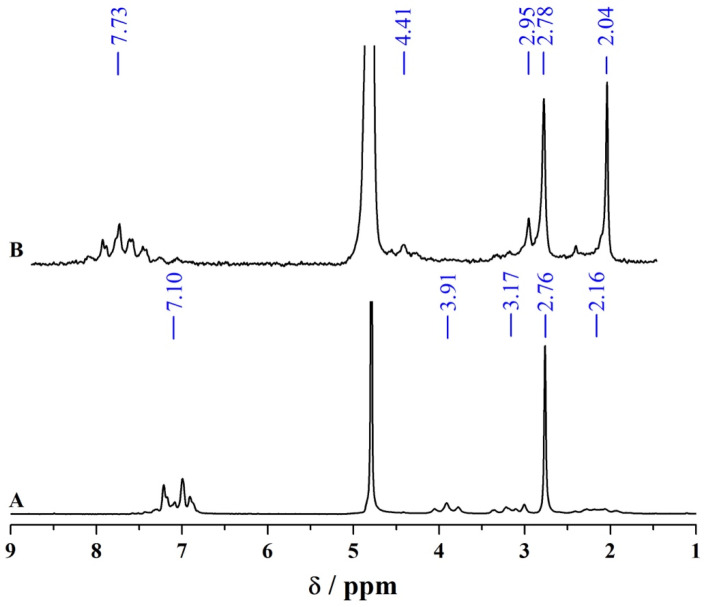
Comparison of ^1^H-NMR spectra in D_2_O before and after electrochemical oxidation of CPZ.HCl: (**A**) ^1^H-NMR spectrum of the untreated CPZ.HCl and (**B**) ^1^H-NMR spectrum of the products obtained following exhaustive controlled-potential bulk electrolysis at *E*_app_ = 1.268 V vs. DmFc^0/+^. Reported chemical shift values correspond to the average (mean) values for multiplets.

^1^H-NMR spectroscopy was employed to determine the chemical structure of the products formed after CPZ electrochemical oxidation and subsequent isolation (Figure 7). In the spectrum of CPZ·HCl before BE (Figure 7A), resonances corresponding to the protonated amine side chain appear at 3.91 ppm (N–C*H*_2_–CH_2_–CH_2_), 3.17 ppm (C*H*_2_–NH(CH_3_)_2_), 2.76 ppm (NH(C*H*_3_)_2_), and 2.16 ppm (CH_2_–C*H*_2_–CH_2_), alongside aromatic multiplets centred at ca. 7.10 ppm.

Following electrochemical oxidation (Figure 7B), two distinct sets of signals emerge, both suggesting sulfoxide (S=O) formation based on the pronounced shift of the aromatic proton signals to around 7.73 ppm, in good agreement with the 7.6–7.8 ppm region reported for phenothiazine sulfoxides in related systems [43]. This is further supported by the significant downfield shift of the methylene protons attached to the PTZ nitrogen (N–C*H*_2_–CH_2_–CH_2_), which move from 3.91 ppm in CPZ·HCl to 4.41 ppm in both oxidation products, reflecting the strong deshielding effect of the electron-withdrawing sulfoxide (S=O) group. Notably, a donative S–O bond typically produces a smaller deshielding effect, causing aromatic proton signals to appear further upfield, often in the 7.40–7.50 ppm region, making its presence unlikely in this context [44]. The upfield shift of the side-chain methyl protons to 2.04 ppm in one set of signals indicates deprotonation of the side-chain amine under isolation conditions. In the second set, an additional alteration in the side-chain environment—including a new proton signal at 2.95 ppm and a methyl signal at 2.78 ppm—supports oxidation at the tertiary amine site, consistent with partial demethylation.

While FT-IR data showed no prominent S=O absorptions in situ, the ^1^H-NMR data indicate the presence of sulfoxide derivatives in the final isolated product. Furthermore, the NMR analysis reveals at least two distinct sulfoxide-containing species. One species retains an unmodified side chain, while the other exhibits partial oxidative demethylation, resulting in a secondary amine.

To further verify the identity of the two oxidation products, BE was carried out at an *E*_app_ of 1.250 V vs. DmFc^0/+^ in CH_3_CN containing 0.1 M [Bu_4_N][PF_6_] as the supporting electrolyte. After BE, a sample was diluted and injected into the LC-MS/MS system for product identification. The final products were identified as chlorpromazine sulfoxide (CPZS=O), with a molecular ion (M^+^) peak observed at 335.0978 *m*/*z*, and nor-chlorpromazine sulfoxide (nor-CPZS=O), characterised by an M^+^ peak at 321.0821 *m*/*z* (Figure S1).

Based on the results presented and supported by previous studies on the homogeneous oxidation of amines [33,34,45,46], the electrochemical oxidation of CPZ.HCl at a fast scan rate (≥0.1 Vs^−1^) is proposed to commence with the oxidation of the phenothiazine nitrogen (Figure 1C, peak Ia), forming the radical cation CPZ.HCl^•+^ (Equation (3)). This is consistent with literature reports and EPR spectroscopy studies, which indicate that the unpaired electron density of the radical cation is primarily localised on the nitrogen atom, with partial distribution across the aromatic ring and sulphur. The orthorhombic symmetry of the spin distribution supports this observation, highlighting significant contributions from nitrogen and sulphur orbitals (Equation (4)) [35,38,39,47,48,49]. Hyperfine coupling constants reveal spin density migration from the nitrogen p-orbital primarily towards the aromatic carbons, stabilised further by solvent interactions [38,39,48,49]. Additionally, SOMO analysis confirms the key role of sulphur in facilitating electron delocalisation, with sulphur p-orbitals playing a dominant role in stabilising the oxidised species [35].(3)
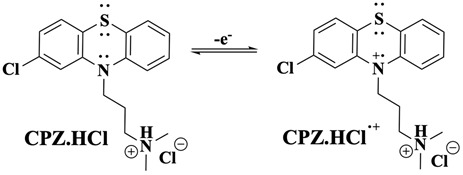
(4)
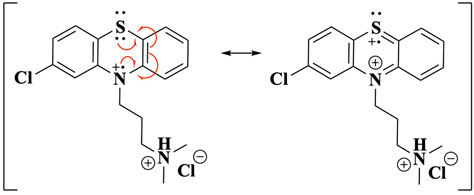


Further oxidation (Figure 1C, peak IIa) results in the loss of a second electron, forming the dication CPZ.HCl^2+^ (Equation (5)). This two-electron oxidation induces a significant structural change in the PTZ moiety of the CPZ molecule, transitioning from a bent to a planar configuration—a consequence of sp^3^ to sp^2^ hybridisation change of nitrogen and sulphur, driven by resonance stabilisation (Equation (6)).(5)
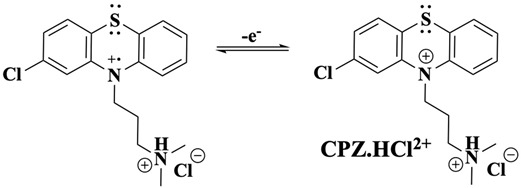
(6)
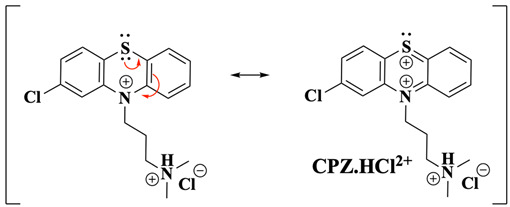


The electrochemical oxidation of CPZ·HCl at slow scan rates begins similarly to the fast scan rate scenario, with the initial oxidation occurring at the nitrogen of the PTZ core, forming the CPZ.HCl^•+^ followed by the formation of CPZ.HCl^2+^ as the potential is scanned to more positive values (Figure 1C, peaks Ia and IIa). However, additional oxidation pathways emerge at these slow scan rates due to the extended timescale, allowing processes with slow kinetic rates to be observed. In this scenario, the side-chain tertiary nitrogen plays a critical role, as it exists in a protonated/deprotonated equilibrium (Equation (7)). This equilibrium directly influences the oxidation kinetics, as only the deprotonated form of the side-chain amine can undergo oxidation. Consequently, the deprotonation kinetics of the amine group in CPZ inherently limit the electron transfer process. The titration study (Figure 3), along with previous findings [34], indicates that the direct oxidation of the side-chain nitrogen occurs at a potential comparable to that of the Cl-PTZ core, justifying the more-than-one-electron transfer observed in the Ψ experiments at slow scan rates (Figure 2). This also aligns with the two-electron oxidation observed during BE at *E*_app_ = 0.950 V, where one electron may correspond to the PTZ nitrogen oxidation and the other to the side-chain tertiary nitrogen oxidation.(7)
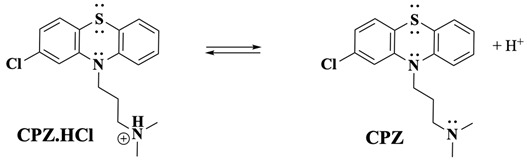


Following oxidation on the side-chain tertiary nitrogen, it undergoes demethylation, a process previously characterised by our laboratory with related tertiary amines [33,34,46]. This reaction is summarised in Equations (8)–(10) (were R = Cl-PTZ), where the intermediate amine radical cation formed after a one-electron oxidation reaction undergoes an α-hydrogen deprotonation to form a terminal methylene radical (Equation (8)), which subsequently disproportionates to yield an amine and an iminium product (Equation (9)). Additionally, a possible disproportionation reaction between CPZ^•+^ (core) and those generated from the one-electron oxidation of the side-chain nitrogen may also occur. The presence of water molecules, whether as impurities in the organic solvent or introduced during extraction, facilitates the reaction of the iminium intermediate to form a secondary amine and formaldehyde (Equation (10)). This secondary amine product becomes electrochemically inactive at the applied BE potential as the nitrogen is transformed into a quaternary nitrogen cation and because the oxidation potential of secondary amines is generally more positive than that of tertiary amines [34], making further oxidation unfavourable under the experimental conditions. These observations are strongly supported by LC-MS/MS and ^1^H-NMR results, which corroborate the presence of nor-CPZS=O as one of the oxidation products.
(8)
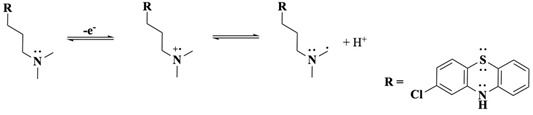

(9)
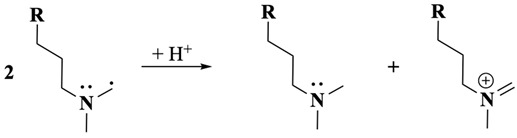

(10)
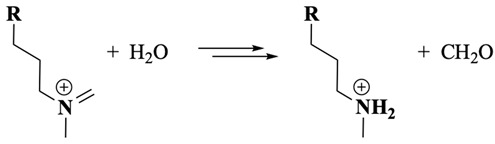


The overall electron transfer count at slow scan rates and during BE experiments accounts for three electrons: two from the PTZ core and one from the side-chain nitrogen. Furthermore, the appearance of CPZS=O and nor-CPZS=O, as detected through ^1^H-NMR and LC-MS/MS analyses, aligns with these observations. Their formation corresponds to the extended reaction times available during slow scans and represents a downstream product of the oxidation sequence.

This suggests that the formation of CPZS=O, observed via ^1^H-NMR and LC-MS analyses, occurs after the completion of the two-electron oxidation process, likely during the final stages of electrolysis or potentially influenced by post-electrolysis handling in the CH_3_CN medium, rather than as an immediate or direct consequence of the oxidation steps as previously stated [25,26]. Spectroelectrochemical UV-Vis and FT-IR studies (Figure 5 and Figure 6) reveal that the CPZ^2+^ structure is notably stable within the experimental timeframe, persisting for approximately 20 min before disappearing. The stability of CPZ^•+^ confirmed through UV-Vis spectroelectrochemical studies at *E*_app_ = 0.950 V vs. DmFc^0/+^ IRS further supports this conclusion.

The incorporation of the S=O group into the CPZ molecule appears to occur gradually over time, facilitated by the extended oxidative conditions present during prolonged BE experiments. This gradual transformation suggests a pathway where the initial oxidation intermediates persist long enough to enable subsequent rearrangements or interactions, leading to the final CPZS=O structure. Environmental factors, including residual water content and solvent interactions, may further promote or stabilise this oxidation step, ultimately enabling the slow incorporation of S=O into the molecule (Equations (11) and (12)). This observation underscores the kinetic nature of S-oxidation, distinguishing it from the faster and more immediate nitrogen oxidation events. For simplicity and better visualisation of the main points, the unbalanced general reaction is presented in Equation (13).(11)
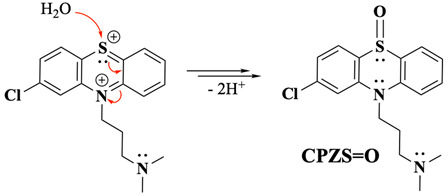
(12)
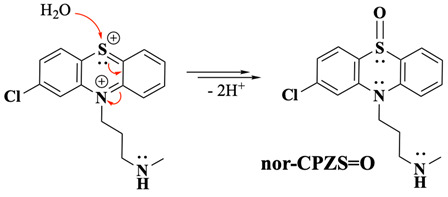


General reaction:



(13)

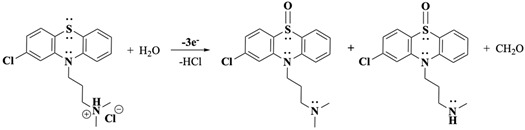




## 3. Materials and Methods

**Reagents and chemicals.** Decamethylferrocene (DmFc, 98%, Sigma-Aldrich, Sydney, Australia), chlorpromazine hydrochloride (CPZ.HCl, ≥98%, Sigma-Aldrich, Sydney, Australia), phenothiazine (PTZ, ≥98%, Sigma-Aldrich, Sydney, Australia), tetrabutylammonium hexafluorophosphate ([Bu_4_N][PF_6_], ≥99%, Sigma-Aldrich, Sydney, Australia), acetonitrile (CH_3_CN, ≥99.9%, Merck, Sydney, Australia), deuterium oxide (D_2_O, 99.9 atom%D, Sigma-Aldrich, Sydney, Australia), dichloromethane (CH_2_Cl_2_, >99%, Fisher Scientific, Melbourne, Australia), ammonium formate (≥99.0%, Sigma-Aldrich, Sydney, Australia), pyridine (≥99.9%, Sigma-Aldrich, Sydney, Australia), nitrogen gas (99.999%, Coregas, Melbourne, Australia), and formic acid (98–100%, Merck, Sydney, Australia), were used as received from the manufacturer.

**Electrochemistry.** Electrochemical experiments were conducted using a Gamry Interface1000 electrochemical workstation (Gamry, Warminster, PA, USA) configured in a standard three-electrode setup. All cyclic voltammetric data were obtained using a 1.0 mm diameter (0.00785 cm^2^ effective area) glassy carbon (GC) working electrode (ALS, Tokyo, Japan). The working electrode area was determined using a standard method [50]. Before each experiment, the electrode was polished with 0.05 µm alumina on a polishing pad (Buehler, Lake Bluff, IL, USA), rinsed with distilled water and acetone, and dried with nitrogen gas or a heat gun. The counter electrode consisted of a coiled platinum wire, while the reference electrode was a double-junction Ag/Ag^+^ electrode (0.01 M AgNO_3_ in CH_3_CN containing 0.1 M [Bu_4_N][PF_6_] as the supporting electrolyte). All electrochemical potentials were referenced to the decamethylferrocene/decamethylferrocenium (DmFc^0/+^) internal reference system (IRS), added after each experiment to avoid analyte interactions.

Experiments were conducted in CH_3_CN with 0.1 M [Bu_4_N][PF_6_] as the supporting electrolyte. For oxygen-free experiments, the solutions were purged with high-purity nitrogen gas for 10 min before measurements, and nitrogen was maintained over the solution to preserve the inert atmosphere throughout the measurements.

Controlled-potential bulk electrolysis (BE) was performed in an H-type electrochemical cell with the working electrode compartment separated from the counter electrode compartment by a fine glass frit. The working electrode was a basket-shaped platinum gauze electrode, and the counter electrode was a reticulated platinum gauze. A platinum gauze electrode was used due to its high conductivity, electrochemical stability, and large surface area, ensuring efficient electron transfer and minimising unwanted side reactions during controlled-potential bulk electrolysis. Controlled-potential bulk electrolysis was conducted at ambient temperature (22 ± 1 °C) in CH_3_CN containing 0.1 M [Bu_4_N][PF_6_]. The reference electrode was the same as that used for cyclic voltammetry experiments. Controlled-potential bulk electrolysis experiments were terminated when the current decreased to less than 1% of its initial value.

Post-electrolysis, the oxidised product was isolated using vacuum drying at 70 °C with a Rotavapor^®^ R-100 (Buchi, Flawil, Switzerland). The separation of the red-coloured product from the supporting electrolyte was achieved via liquid–liquid extraction using a 1:1 MilliQ water–CH_2_Cl_2_ mixture. The red-coloured aqueous phase was subsequently dried by vacuum at 90 °C using a Rotavapor.

**Spectroscopy.** Spectroelectrochemical UV-Vis data obtained from the oxidation of CPZ in CH_3_CN containing 0.1 M [Bu_4_N][PF_6_] as the supporting electrolyte were recorded using a 2.0 mm optical path length rectangular quartz cuvette (ALS, Tokyo, Japan), a platinum gauze working electrode, a platinum wire auxiliary electrode, and an Ag/Ag^+^, 0.1 M [Bu_4_N][PF_6_] double-junction pseudo-reference electrode. The UV-Vis spectra were monitored throughout the BE process, using a USB4000XN spectrometer (Ocean Optics, Inc., Orlando, FL, USA) at wavelengths from 200 to 900 nm until the current had decreased to less than 1% of its initial value.

The FT-IR spectroscopy was used to investigate the chemical changes during CPZ oxidation. Samples were collected at various intervals during BE, deposited on KBr disks, and dried to remove solvent. Spectra were recorded with an FTS3000 Excalibur Series FT-IR Infrared Spectrometer (BioRad, Hercules, CA, USA) at a resolution of 4 cm^−1^.

Proton (^1^H)-NMR spectroscopy measurements were performed on a Spinsolve 43 MHz Benchtop NMR Spectrometer (Magritek, Wellington, New Zealand). The BE product samples were reconstituted in D_2_O, and chemical shifts were referenced to the residual water resonance at 4.79 ppm.

Liquid chromatography coupled to an ultra-high-resolution mass spectrometer (Vanquish Flex UHPLC coupled with an Orbitrap Exploris-240, Thermo Fisher Scientific, Waltham, MA, USA) was also employed to analyse BE products. Chromatographic separation was achieved using a 2.1 × 50 mm, 2.7 μm Agilent Poroshell SB-C18 column, maintained at 30 °C. The mobile phase consisted of 0.1% formic acid in water (mobile phase A) and 0.1% formic acid in CH_3_CN (mobile phase B). Gradient elution was performed at a flow rate of 0.4 mL min^−1^, starting at 5% B and increasing linearly over 7 min to 100% B, which was maintained for 3 min. The column was then re-equilibrated at 5% B for 3 min, resulting in a total run time of 13 min. For MS analysis, data-dependent MS/MS mode was used, with electrospray ionisation (ESI) in positive mode. The full MS scan was performed at a resolution of 120,000 over the *m*/*z* range of 70–1000, while the MS/MS scans were conducted at a resolution of 15,000. The full scan and MS/MS acquisition time was 0.6 cycles per second, and normalised collision energies of 30, 50, and 70 were applied for MS/MS fragmentation. A 50,000-count threshold was set for MS/MS fragmentation to ensure signal sensitivity.

## 4. Conclusions

This study provides a comprehensive mechanistic investigation into the electrochemical oxidation of CPZ, revealing critical insights beyond the conventional focus on the PTZ core. Our findings establish a three-electron oxidation pathway, challenging the historically accepted two-electron paradigm and offering a refined perspective on CPZ redox behaviour in non-aqueous systems.

Through an integrated experimental approach, we demonstrate that the oxidation sequence involves not only the PTZ core but also significant contributions from the tertiary amine side chain. This side-chain oxidation, leading to the formation of nor-CPZ sulfoxide, has often been overlooked in previous studies. Our results highlight the critical role of structural rearrangements in dictating the fate of CPZ oxidation products, with spectroelectrochemical evidence confirming the existence of stable intermediate species.

While these findings are specific to acetonitrile, they offer valuable mechanistic insights that could aid the broader interpretation of CPZ oxidation pathways. By elucidating these fundamental electrochemical transformations, this study provides a framework for future studies exploring CPZ oxidation under different solvent conditions, including aqueous environments more relevant to pharmacological metabolism. Ultimately, this work refines our understanding of CPZ redox chemistry and lays the groundwork for further research into its structural and electronic properties.

## Data Availability

Data will be made available on request.

## Supplementary Materials

The following supporting information can be downloaded at: https://www.mdpi.com/article/10.3390/molecules30051050/s1, Figure S1: LC-MS/MS analysis of oxidation products from CPZ before and after BE.
