# Supplementary material for: Beyond the Phenothiazine Core: Mechanistic Insights into the Three-Electron Oxidation of Chlorpromazine
Source: Molecules. 2025 Feb 25;30(5):1050. doi: 10.3390/molecules30051050 (PMC11901959; doi:10.3390/molecules30051050)
Supplement: Supplementary file 1 [file molecules-30-01050-s001.zip › molecules-3490837-supplementary.pdf]

Supporting Information

Mechanistic Insights into the Electrochemical Oxidation of Chlorpromazine: Beyond the Phenothiazine Core

Kiara T. Miller, Ashwin K. V. Mruthunjaya, Angel A. J. Torriero*

School of Life and Environmental Sciences, Faculty of Science Engineering & Built Environment, Deakin University, Burwood, VIC 3125, Australia

* Corresponding author: E-mail address: angel.torriero@deakin.edu.au.

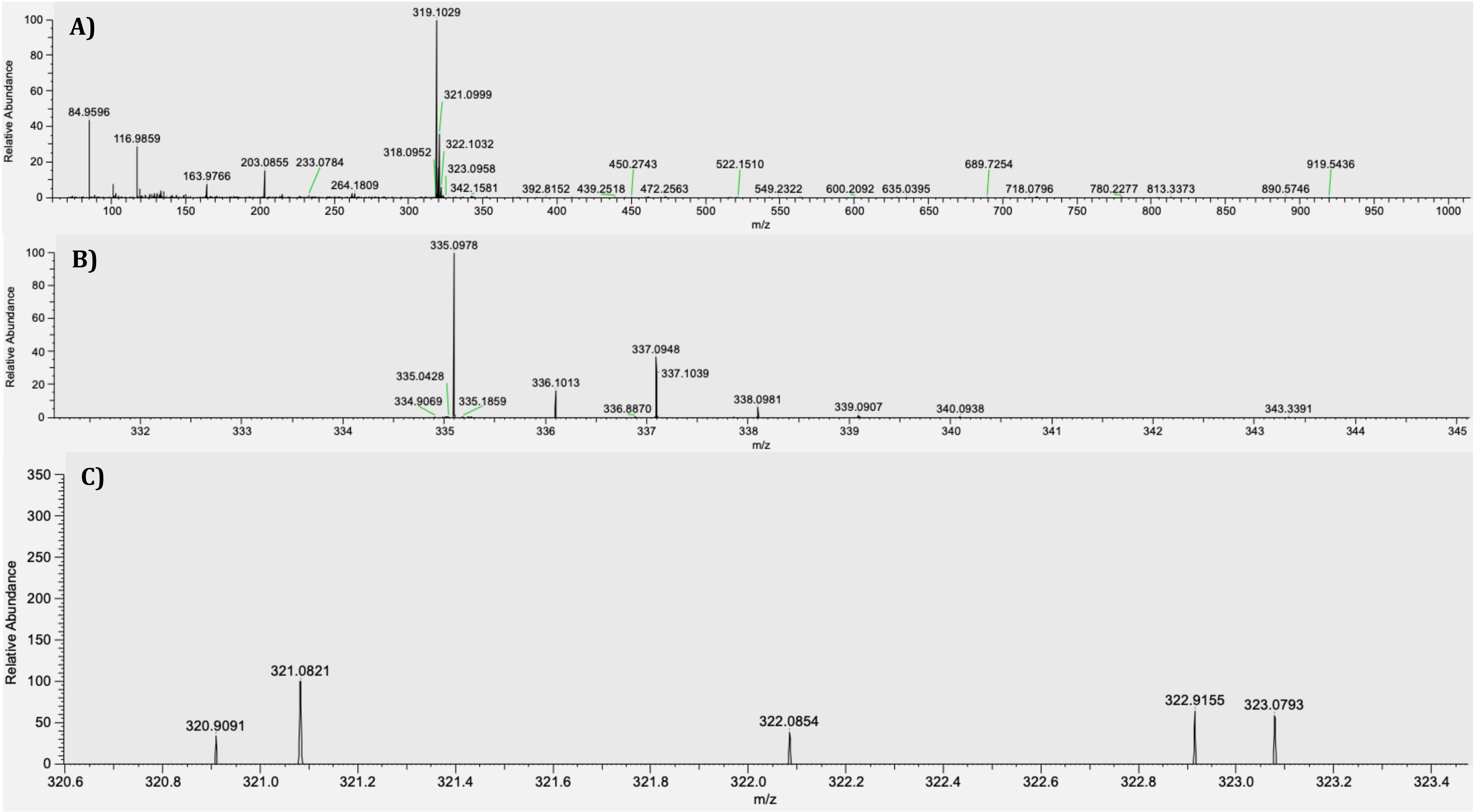

Figure S1. LC-MS/MS analysis of oxidation products from CPZ before and after BE. **A)** Mass spectrum of the starting CPZ material; **B)** Enlarged view highlighting the molecular ion (M^+) peak at 335.0978 m/z, corresponding to chlorpromazine sulfoxide (CPZS=O); **C)** Enlarged view showing the M^+ peak at 321.0821 m/z, corresponding to nor-chlorpromazine sulfoxide (nor-CPZS=O).
